# Safety and Efficacy of Intranasal Ketamine for Minor Pediatric Procedures: A Systemic Literature Review

**DOI:** 10.7759/cureus.62605

**Published:** 2024-06-18

**Authors:** Yasir S Alkhalifah

**Affiliations:** 1 Department of Pediatrics, College of Medicine, Qassim University, Buraidah, SAU

**Keywords:** systematic review, pediatric, sedation, ik, intranasal ketamine

## Abstract

Compared to intravenous anesthetics, intranasal medication for sedation is a less invasive approach. Intranasal ketamine (IK) is a widely used medication for procedural sedation. Hence, a systematic review was conducted with the aim of evaluating the safety and efficacy of IK among pediatric patients used for procedural sedation. For this purpose, a literature search was conducted on PubMed, Web of Science, and MEDLINE. A total of 247 search results appeared after running the developed query and eight articles passed through the inclusion and exclusion criteria and were included in the study. Most of the studies used 4 to 7 mg/kg dose of IK for pediatric patients. It was found that there was a moderate correlation between the age and dose of IK. Similarly, the dose of IK also had a direct and moderate correlation with the onset of sedation. Regarding the reported side effects, most of the studies reported nausea and vomiting as side effects of IK. Hence, it could be concluded from the study findings that effective sedation can be achieved by using 4 to 7 mg/kg dose of IK. The reported side effects of IK were minor while parental satisfaction with the drug was very high. Therefore, it can be concluded that the use of IK for procedural sedation among pediatric patients is safe and effective.

## Introduction and background

Every day, several painful or anxiety-provoking procedures are performed on pediatric patients. For example, cases presented in the emergency department include laceration repair, abscess incision and drainage, fracture reduction, and foreign body removal. Similarly, during magnetic resonance imaging (MRI) or another type of radiographic procedure, a patient must remain calm and motionless. The interpretation of intricate dynamic anatomy and pathological presentation requires images of optimal quality [1].

Historically, these procedures involved using force to restrain the child. Because of this method, children had to endure a great deal of pain and trauma. Furthermore, their unwillingness to cooperate may have harmed the quality of the treatment or procedure [2]. To address the issue, anxiolytic and sedative medications have been used during these procedures to improve procedural performance and patient comfort [3].

Furthermore, children have a higher fear of physicians, injections, and operating rooms than adults [4]. Furthermore, the feeling of being separated from parents and being among completely unfamiliar faces makes the treatment process more traumatic for young children. Furthermore, negative responses to future visits and post-traumatic stress symptoms were reported if the procedure was not accompanied by adequate pain control and anxiolysis [5]. Furthermore, children who experienced high anxiety prior to the procedure exhibited negative post-procedural behaviors such as sleep anxiety, separation anxiety, aggression, and so on [6].

These issues can be alleviated with traumatic sedation. Intranasal (IN) drug delivery is less invasive and more convenient than canula or intramuscular injections [7]. However, IN and intramuscular administration allow for less control of drug levels/depth and duration of sedation. Intranasal ketamine (IK) is used to induce sedation and analgesia in pediatric patients undergoing non-operative procedures in the emergency department, oncology, dentistry, radiation therapy, and radiology [8]. Besides ketamine, another widely used sedative medication is midazolam. For the preanesthetic medication for pediatric patients, midazolam is the most frequently used medication [9]. However, several studies have reported the efficacy of IK in pain relief and effective sedation with the least side effects [10,11].

This systematic review will include all studies that discussed minor pediatric procedures such as laceration, IV canulation, MRI, radiography, and ultrasound while using IK for sedation. This systematic review will look at the safety and efficacy of IK. The study also aimed to determine the appropriate IK dosage for procedural sedation. Additionally, any reported adverse event or side effect will be extracted from the included studies.

## Review

Methods

Study Design and Search Strategy 

In May 2024, a comprehensive literature search was carried out for this retrospective study. This systematic review was conducted in accordance with the Preferred Reporting Items for Systematic Reviews and Meta-Analysis (PRISMA). The search engines used in this literature search were MEDLINE, SCOPUS, and Web of Science. Initially, there were no language or data restrictions.

The medical subject heading (MeSH) terms used for the literature search were as follows: (“Intranasal Ketamine” OR “Nasal Ketamine”, “Ketamine Nasal Spray”, “Intranasal Esketamine”, “Nasal Route Ketamine”, “Pediatric”, “Paediatric”, “Child”, “Children”, “Pædiatric”

Eligibility Criteria

The first search yielded 247 studies. After removing duplicate records, 125 studies were left out. An Excel file was created which included the 125 studies, their titles, a list of authors, author keywords, and abstracts.

**Figure 1:**
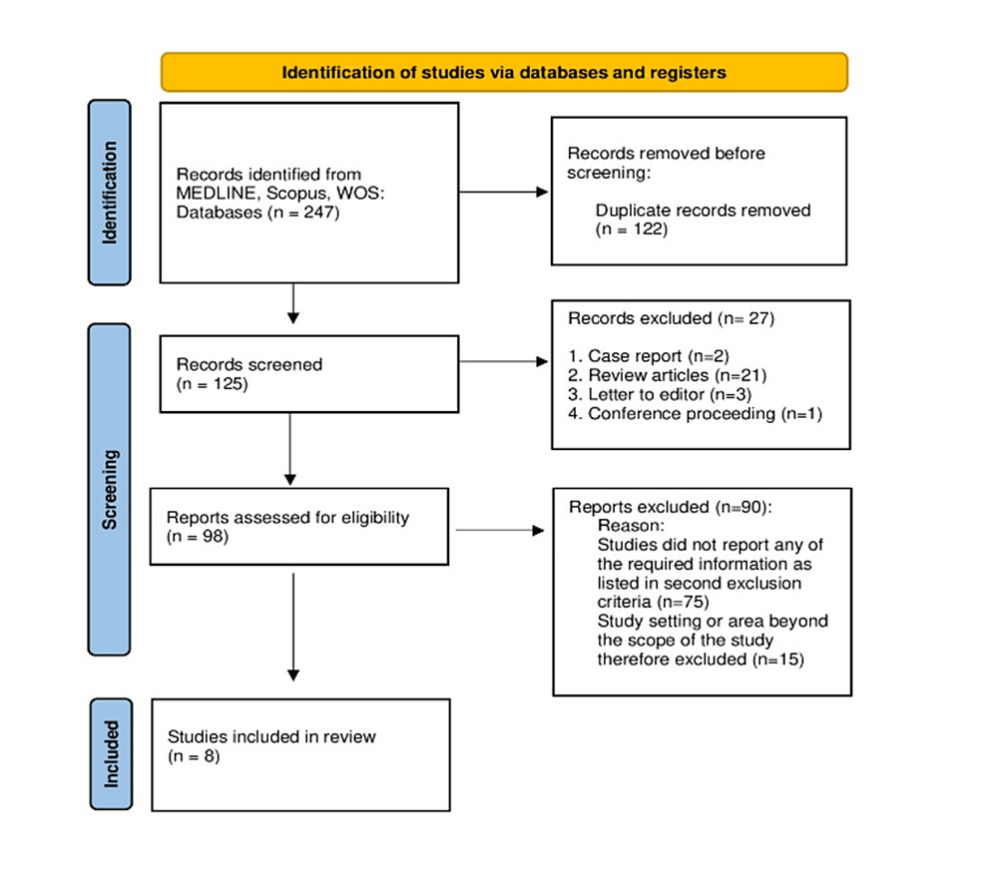
PRISMA flow diagram of the selection of studies for inclusion in the systematic review PRISMA: Preferred Reporting Items for Systematic Reviews and Meta-Analysis

In the first screening, the type of article was determined by reviewing the study titles, and it was discovered that two records were case reports, 21 were review articles, three were letters to the editor, and one was a conference proceeding. Thus, 98 studies remained to be screened further. In the second screening, the remaining 98 studies' titles, abstracts, and keywords were examined. Studies that did not use IK for sedation or report on the safety and efficacy of IK among pediatric patients were excluded from the second screening (n=75). By looking at the abstracts, the remaining 23 studies were screened for the setting or specialty. It was discovered that 12 of the studies dealt with surgery, four with emergency rooms, five with radiology, one with dentistry, and one with bone marrow biopsy cases. Nonetheless, studies on emergency and radiology departments had to be chosen for data extraction in accordance with the study's objectives. Eight studies remained for the data extraction process after one radiology-related study that described the use of IK for radiation therapy was also eliminated (Figure 1).

Data Extraction

The data was extracted for the articles passed through the inclusion criteria and tabulated in Table 1. The data extracted consisted of the following variables: age, gender, sample size, dose of IK, onset of sedation, sedation score, adverse event, complication and parental satisfaction.

**Table 1:** Descriptive data and IK dosage reported in the studies M: Male; F: female, mg/kg: milligram/kilogram; IK: intranasal ketamine

Author, year	Average/median age (years)	Age interval (years)	Gender	Sample size	Dose
Rached-d'Astous et al., 2023 [12]	3.2 (median)	1-12	M=22, F=8	30	6 mg/kg
Tsze et al., 2012 [13]	-	1-7	-	12	3-9 mg/kg (adequate sedation got at 9 mg/kg)
Cristoforo et al., 2022 [14]	5.1 (mean)	≥2	M=17, F=8	25	5 mg/kg
Jafarnejad et al., 2020 [15]	5.22 (mean)	2-8	M=19, F=16	35	5 mg/kg
Liu et al., 2021 [16]	3.4(mean)	1-6	M=75, F=93	168	2 mg/kg
Gyanesh et al., 2014 [17]	4.9(mean)	1-10	M=36, F=17	52	5 mg/kg
Ibrahim, 2014 [18]	7.12(mean)	4-10	M=14, F=15	29	7 mg/kg
Alp et al., 2019 [19]	2 (median)	≤3	M=29, F=43	72	4 mg/kg

Results

As illustrated in Figure 1, there were eight studies that were passed through the inclusion and exclusion criteria and included in the present systematic review for data extraction. The demographic data (age and gender), sample size and dose of IK provided to the patients were tabulated in Table 1. Among all the included studies, only one study had an age interval up to 12 years; however, other studies had a sample population maximum of up to 10 years. The sum of the sample size extracted from the studies was 458 in which there were 212 (51.5%) males and 200 (48.5%) females. Hence, the proportion of male patients was slightly higher than that of females. Regarding the dosage of IK, it was at least 4 mg/kg or more. There were only two studies that used less than 5 mg/kg dose of IK for sedation. The correlation between the average/median age and the given dose of IK was tested. It was found that the correlation coefficient was 0.52 which showed a moderate and direct correlation between the age of a patient and dose of IK however the correlation was not statistically significant (p=0.233).

The onset of sedation was reported in five out of eight studies. The minimum reported onset of sedation time was 6.8 minutes while the longest time taken was 34 minutes (Table 2). Similarly, the minimum sedation duration was reported to be 36 minutes while the maximum sedation duration was 55 minutes. Studies also reported the number and percentage of participants for whom sedation was found effective. Only one study reported the low effectiveness of sedation (25%) while most of the studies (6 out of 8) found sedation effective for at least 80% of the study sample (Table 2). Partial correlation was used, in which age was used as a controlling factor, to test the correlation between dose and sedation onset, effectiveness, and duration. The correlation coefficient was 0.537 (p=0.5) and showed a direct, moderate, and insignificant correlation between dose and sedation onset. Similarly, correlation between dose and sedation effectiveness was -0.445 (p=0.376) which showed inverse, moderate, and insignificant correlation. However, the partial correlation test between dose and sedation duration was not run because of limited data.

**Table 2:** Sedation characteristics of the intranasal ketamine mg/kg: milligram/kilogram, Min: minutes, UMSS: University of Michigan Sedation Scale, RASS: Richmond Agitation Sedation Scale, OSBD-R: observational scale of behavioral distress-revised

Author, year	Dose	Sedation onset	Effectiveness of sedation	Sedation duration	Sedation status	Sedation efficacy
Rached-d'Astous et al., 2023 [12]	6 mg/kg	18 min (median)	18 (60%)	49 min (median)	UMSS score: 15(minimally sedated, 6(moderately sedated), 3(deeply sedated), 6(UMSS=0, awake and alert)	-
Tsze et al., 2012 [13]	3-9 mg/kg (adequate sedation got at 9 mg/kg)	6.8 min (median)	3 (25%)	36 min (median)	-	OSBD-R = 0.34(0.38)
Cristoforo et al., 2022 [14]	5 mg/kg	-	20(80%)	-	According to calm or drowsy rating. 11 stayed calm, 9 drowsy, 5 not sedated	-
Jafarnejad et al., 2020 [15]	5 mg/kg	-	29(82.9%)	-	-	OSBD-R= 3.51(SD=1.33)
Liu et al., 2021 [16]	2 mg/kg	10.9 min (mean)	138(82.1%)	53.8min (SD15.2)	30 unsuccessful sedations. 6 failed to achieve adequate sedation, 9 awakened or moved, 15 unconscious body movement	-
Gyanesh et al., 2014 [17]	5 mg/kg	-	43(82.7%)	-	-	-
Ibrahim, 2014 [18]	7 mg/kg	14.65min (mean)	23(79.4%)	-	-	Ramsay sedation scale, 4.58(SD0.9)
Alp et al., 2019 [19]	4 mg/kg	34 min (median)	69(95.8%)	55 min	According to the RASS scale after 30 mins, 51 were drowsy, 18 sedated	RASS score (n=18, 26.1%) after 30 mins

Reported side effects either during or after the procedure were extracted from the studies. In addition, adverse events and parental and physician satisfaction were also extracted from the included studies (Table 3). All eight studies reported the side effects of IK in which vomiting and nausea were the most common prevalent side effects and reported in all eight studies. In addition, dizziness, desaturation, and hiccups were other reported side effects but it was not reported by more than one study. Adverse events were only reported in one study in which airway obstruction, emergency agitation, and delayed awakening were the reported adverse events. Contrary to the side effects of the IK, parental satisfaction and future use of IK if required was as high as 78.6% to 92.4%. There were four studies that reported parental satisfaction while three studies reported physician satisfaction. Physician satisfaction was also as high as 75% to 84% (Table 3).

**Table 3:** Intranasal ketamine side effects, adverse events, and satisfaction

Author, year	Side effects during or after procedure	Adverse event	Parental satisfaction	Physician satisfaction
Rached-d'Astous et al., 2023 [12]	4(13%) (vomiting, nausea, dizziness), 2(7%) (desaturation)	0	25(89%)	21(75%)
Tsze et al., 2012 [13]	1(33.3%) (vomiting)	0	-	-
Cristoforo et al., 2022 [14]	2(8%) bad taste, 2(8%) (vomiting, nausea, nystagmus), 1(4%) dizziness	0	21(84%)	21(84%)
Jafarnejad et al., 2020 [15]	18(51.4%) nausea and vomiting, 1(2.9%) hiccups	0	-	-
Liu et al., 2021 [16]	2(1.2%) vomiting	2(1.2%) airway obstruction, 2(1.2%) emergency agitation, 2(1.2%) delayed awakening	132(78.6%)	
Gyanesh et al., 2014 [17]	5(9.6%) nausea and vomiting	-	92.4%	82.7%
Ibrahim, 2014 [18]	5(17.2%) nausea and vomiting	-	-	-
Alp et al., 2019 [19]	2(2.9%) nausea and vomiting	-	-	-

Discussion

The purpose of this systematic review was to assess the safety and efficacy of IK among pediatric patients who visited the emergency or radiology departments. The current systematic review included all published studies with no time limitations. Inclusion and exclusion criteria were applied to the searched articles, and eight studies were selected and included in the current systemic review. Figure 1 depicts the PRISMA flow chart diagram, which provides details and explanations for the application of inclusion and exclusion criteria.

Descriptive analysis of the dose of IK used for procedural sedation revealed that the minimum dose given to pediatric patients was 2 mg/kg [16]. The included patients ranged in age from 1 to 6 years, and 82% of them reported sedation effectiveness [16]. In contrast, Alp et al. published a study in which patients as young as three years old were given a dose of IK of 4 mg/kg [19]. Effective sedation was achieved in as many as 96% of the study participants (Table 2). As a result, the correlation between age and IK dose was calculated, and it was found to be moderate. The correlation coefficient revealed that an increase in the IK dose was proportional to the patients' age. However, the finding was not significant, which could be attributed to the sample size of the input data used to compute the correlation. The doses used in the published studies ranged from 4 to 7 mg/kg. However, Liu et al. used 2 mg/kg, and Tsze et al. used 9 mg/kg IK for sedation, which could be considered outliers [13,16]. Several other studies have been published to determine the dose of IK, and it has been reported that a dose of 5 to 6 mg/kg can provide adequate sedation [20-22].

Aside from the sedation efficacy reported in published studies, the efficacy of IK for procedural sedation could be determined based on sedation effectiveness. The reason for using reported sedation effectiveness in addition to sedation efficacy was that there were only a few studies that reported sedation efficacy. Four out of eight studies reported sedation efficacy. In addition, various scales were used to assess efficacy. The scales used were OSBD-R, Ramsay sedation scale, and RASS (see Table 2). Tsze et al. and Jafarnejad et al. used the OSBD-R scale. Tsze et al. reported an average sedation efficacy score of 0.34, whereas Jafarnejad et al. reported 3.51 [13,15]. The low score reported by Tsze et al. could be attributed to the sample size used in the study. The study began with 12 patients, but only three met the inclusion criteria [13]. Ibrahim used the Ramsey sedation scale, and the average sedation efficacy was 4.58 [18]. Sedation efficacy was determined by the RASS score in 26% of patients after 30 minutes of the dose administered [19]. The inverse correlation between sedation effectiveness and the IK dose demonstrated that increasing the dose would necessarily increase sedation effectiveness. Review of the extracted data showed that the studies achieved effective sedation at least among 80% of study sample were mostly used 4 to 5 mg/kg of IK dose.

Only one of the eight studies reported an adverse event after using IK for sedation purposes. Liu et al. published a study with 168 patients, six of whom (3.6%) experienced adverse events. However, studies found that the most common side effects were vomiting and nausea. Traditionally, perioperative use of inhalational anesthesia was associated with nausea/vomiting and agitation [23,24]. However, some researchers disagree with the hypothesis that premedication is effective in reducing side effects [25-27].

Lang et al. published a meta-analysis that found that administering IK to pediatric patients could cause severe changes in hemodynamic parameters such as blood pressure and heart rate [28,29]. Furthermore, it was reported that cardiovascular effects occurred frequently during or immediately after ketamine intravenous administration [30]. As a result, nasal administration of ketamine is safer, as few studies included in the current systematic review reported adverse effects. Most studies reported minor side effects such as nausea and vomiting. Apart from the minor side effects, parental satisfaction with the use of IK for procedural sedation was extremely high. In addition, three studies included physicians' satisfaction and reported a percentage of 75% to 84% who were satisfied with the use of IK for sedation. Moreover, administration of IK in radiology, emergency or any other department did not vary the parental or physician satisfaction level significantly and a high satisfaction towards the drug was observed.

Most of the studies included in this systematic review had small sample sizes, so their findings could not be conclusive or comprehensive, which could compromise the generalizability of this systematic review. Another limitation was the use of only ketamine in the study; comparing ketamine to any other drug would help to highlight the superiority of one over the other. In addition, the present study did not include the studies that used IV dose of ketamine hence comparison of effect of IV vs IN could not be performed. Moreover, the method of IN administration of medication (atomized, nebulized or sprayed) was also not noted because it was out of the scope of the study. 

## Conclusions

This systematic review assessed the safety and efficacy of using IK for procedural sedation. Effective sedation was achieved with 4 to 7 mg/kg of IK. Giving IK doses in this range can provide effective sedation, and an acceptable sedation efficacy score was reported. Furthermore, the most reported side effects of IK were vomiting and nausea, with no severe side effects or adverse events reported. Furthermore, parent satisfaction with the use of IK was high. As a result, this study demonstrated that using IK for procedural sedation in pediatric patients is both safe and effective.
